# Does Cognitive Status Moderate the Relationship between Environmental Factors and Self-Reported Health?

**DOI:** 10.1093/geroni/igab046.2657

**Published:** 2021-12-17

**Authors:** Ethan Siu Leung Cheung, Ada Mui

**Affiliations:** 1 Columbia University, New York, New York, United States; 2 Columbia University, COLUMBIA UNIVERSITY, New York, United States

## Abstract

Using data from NHATS Round 9, the present study examines the relationships between environmental factors and self-reported health among older adults with dementia, mild cognitive impairment (MCI), and normal cognition. Based on neighborhood stress process theory, we investigate the following questions: 1) Are there associations between dwelling safety hazards and neighborhood environments and self-reported health? 2) Is cognitive status a moderator between the relationship? 3) How do these associations differ between older adults with varying cognitive status (i.e., dementia, MCI, and normal cognition)? A hierarchical linear regression analyses are conducted. Results indicate that better quality of sidewalk surface and neighborhood social cohesion are associated with better self-reported health, after taking into account sociodemographic, health, and social factors. Interaction terms are then used to examine the moderating effects of cognitive status on the associations; four interactions terms are found to be statistically significant. Lastly, separate linear regression analyses are implemented for the dementia, MCI, and normal cognition groups. Findings show that the predicting power of environmental factors vary by cognitive status of older adults. For individuals with dementia, tripping hazards, cluttered home, and community disconnectedness are associated with poor self-reported health. However, no significant relationship was found for older adults with MCI. For older adults with normal cognition, better quality of sidewalk surface and neighborhood social cohesion predict better self-rated health scores. Findings of this study illuminate the important role of a hazard-free home, community walkability, and socially cohesive neighborhood environments in predicting better health status of older adults.

